# A two-phase bromination process using tetraalkylammonium hydroxide for the practical synthesis of α-bromolactones from lactones

**DOI:** 10.3762/bjoc.17.198

**Published:** 2021-12-09

**Authors:** Yuki Yamamoto, Akihiro Tabuchi, Kazumi Hosono, Takanori Ochi, Kento Yamazaki, Shintaro Kodama, Akihiro Nomoto, Akiya Ogawa

**Affiliations:** 1 Department of Applied Chemistry, Graduate School of Engineering, Osaka Prefecture University, 1-1 Gakuen-cho, Nakaku, Sakai, Osaka 599-8531, Japan; 2 Nippoh Chemicals Co., Ltd. Neo Kawai Building, 8-15,4-Chome, Nihonbashi-Honchou, Chuo-Ku, Tokyo 103-0023, Japan

**Keywords:** α-bromolactone, metal-free, one-pot operation, tetraalkylammonium hydroxide, two-phase system

## Abstract

A simple and efficient method for α-brominating lactones that affords α*-*bromolactones under mild conditions using tetraalkylammonium hydroxide (R_4_N^+^OH^−^) as a base was developed. Lactones are ring-opened with Br_2_ and a substoichiometric amount of PBr_3_, leading to good yields of the corresponding α-bromocarboxylic acids. Subsequent intramolecular cyclization over 1 h using a two-phase system (H_2_O/CHCl_3_) containing R_4_N^+^OH^−^ afforded α-bromo lactones in good yields. This method can be applied at the 10 mmol scale using simple operations. α-Bromo-δ-valerolactone, which is extremely reactive and difficult to isolate, could be isolated and stored in a freezer for about one week using the developed method. Optimizing the solvent for environmentally friendly large-scale syntheses revealed that methyl ethyl ketone (MEK) was as effective. In addition, in situ-generated α-bromo-δ-valerolactone was directly converted into a sulfur-substituted functional lactone without difficulty by reacting it with a sulfur nucleophile in one pot without isolation. This new bromination system is expected to facilitate the industrial use of α-bromolactones as important intermediates.

## Introduction

Lactones are important heterocycles in the organic chemistry, materials science, and medicinal chemistry fields, and bromolactones are important synthetic intermediates for selectively, effectively, and practically introducing lactone units into organic molecules [1–18]. Among brominated lactones, the α-bromolactone, in which the bromine atom is located at the α-position relative to the carbonyl group, is the most versatile synthetic intermediate [19–28]. α-Bromolactones are widely used as synthetic intermediates for functional materials and pharmaceuticals, as well as initiators in atom-transfer living radical polymerization (ATRP) reactions and functional polymer synthesis [29–34].

**Scheme 1:**
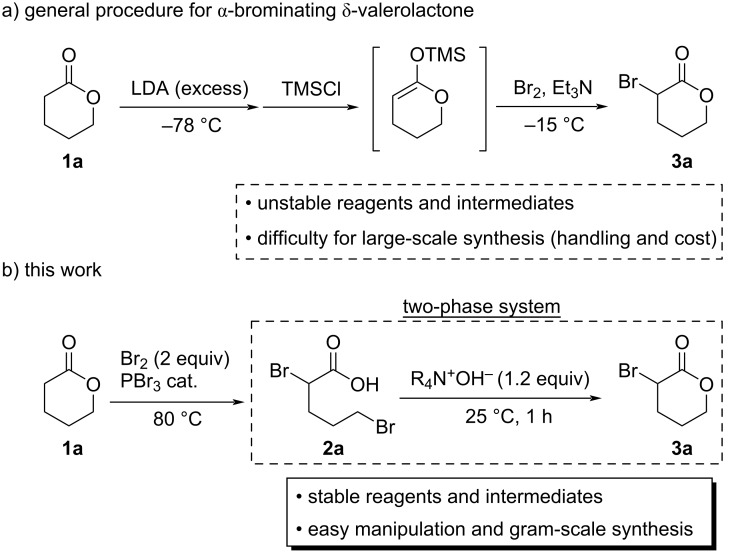
General procedure for *α*-bromination of δ-valerolactone (**1a**) and the method described in this work.

Although α-bromo-γ-butyrolactone, which is a five-membered lactone, is easily accessible from the five-membered lactone by some bromination methods [35–36], the bromination method for the six-membered lactone, δ-valerolactone (**1a**), has been limited. The main process for the bromination of δ-valerolactone (**1a**) was treating the lactone with lithium diisopropylamide (LDA) at −78 °C to first generate the corresponding enolate, trapping it with trimethylsilyl chloride (TMSCl) to form the enol silyl ether, followed by reaction with bromine (Scheme 1a) [37–38]. While the industrial demand for α-bromolactones has grown in recent years, the above-mentioned laboratory-level synthetic methods are not suitable for scale-up because LDA, TMSCl, and enol silyl ethers are sensitive to moisture and air, as well as being expensive for large-scale syntheses. Therefore, the development of an innovative, cost-effective method for the production of α-bromolactones in large quantities is highly desirable.

In this study, we aimed to establish a new method for the synthesis of α-bromolactones and successfully developed an innovative synthetic method that relies on a two-phase reaction system. Specifically, lactones are ring-opened and converted into dibromocarboxylic acids when treated with Br_2_ and a substoichiometric amount of PBr_3_. Subsequent treatment of these carboxylic acids with a base leads to the corresponding α-bromolactones through ring-closing reactions that involve the elimination of HBr; notably, ring-closure is successfully promoted in a two-phase system (Scheme 1b). Furthermore, we also report the simple one-pot transformations of lactone derivatives using α-bromolactones as key intermediates.

## Results and Discussion

We begin by first discussing the properties and stabilities of industrially important five- and six-membered lactones. γ-Butyrolactone and its α-brominated derivative are both stable at room temperature; α-bromo-γ-butyrolactone is readily synthesized by brominating the five-membered lactone under basic conditions. In sharp contrast, the corresponding six-membered α-bromo-δ-valerolactone has a more-distorted ring and is extremely unstable, even at room temperature [39–41]. In fact, it must be stored in a freezer because ring-opening polymerization and ring-contraction reactions occur readily at room temperature (see Supporting Information File 1). Therefore, in this study, we chose unstable δ-valerolactone (**1a**) as a model compound during the development of a new and innovative method for the synthesis of α-bromolactones, and investigated the reaction conditions in detail.

We first examined the Hell–Volhard–Zelinsky-type ring-opening reaction of **1a** (Table 1). In this reaction, the corresponding acid bromide is formed in situ by heating with Br_2_ and a substoichiometric amount of PBr_3_; the acid bromide is then converted into 2,5-dibromopentanoic acid (**2a**) via hydrolysis during the workup under open-air. Lactone **1a** (5 mmol) was allowed to react with Br_2_ (2.0 equiv) and PBr_3_ (5 mol %) at 80 °C for 24 h, with subsequent hydrolysis successfully affording in **2a** in 90% yield (Table 1, entry 1). In the absence of a substoichiometric amount of PBr_3_, the transformation of **1a** to **2a** hardly proceeded (Table 1, entry 2). **2a** was obtained in 89% yield when this protocol was used on a 31 mmol scale (Table 1, entry 3). γ-Butyrolactone (**1b**) and ε-caprolactone (**1c**) were also converted into the corresponding dibromocarboxylic acids **2b** and **2c** in yields of 76% and 70%, respectively (Table 1, entries 4 and 5).

**Table 1:** Ring-opening reactions of lactones with Br_2_ in the presence of a substoichiometric amount of PBr_3_^a^.

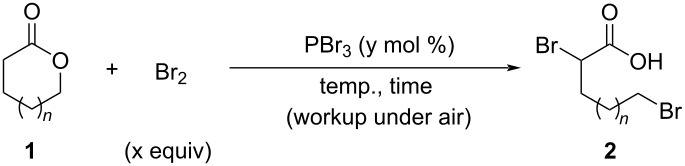							

Entry	*n*	**1** (mmol)	Br_2_ (equiv)	PBr_3_ (mol %)	Temp. (°C)	Time (h)	Yield **2** (%)

1	**1a**: *n* = 1	5	2.0	5	80	24	**2a**: 90
2	**1a**: *n* = 1	5	2.0	–	80	24	**2a**: 1
3	**1a**: *n* = 1	31	2.0	10	80	24	**2a**: 89
4	**1b**: *n* = 0	31	2.0	10	90	24	**2b**: 76
5	**1c**: *n* = 2	31	2.0	10	90	24	**2c**: 70

^a^Yields were determined by ^1^H NMR spectroscopy using 1,3,5-trioxane as an internal standard.

**Scheme 2:**
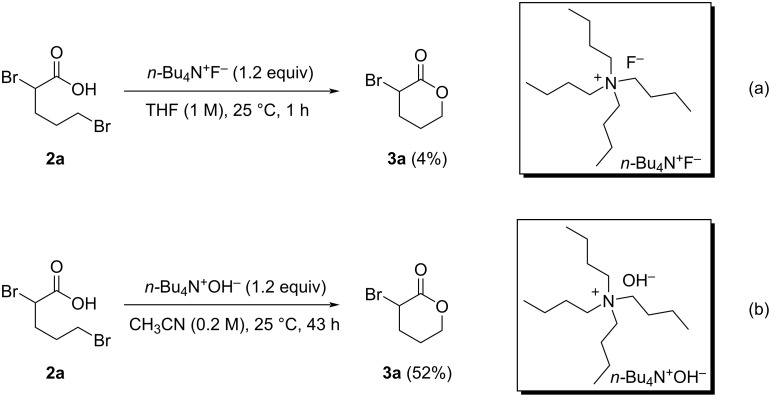
Tetraalkylammonium salt-mediated intramolecular cyclization of **2a**.

Since we successfully synthesized carboxylic acid **2a** from lactone **1a** in good yield, we next investigated the ring-closing reaction of **2a**. Various acids and bases (PTSA, hydrochloric acid, NaOH, KOH, and NaHCO_3_) were used to promote the intramolecular cyclization of **2a**; however, no reaction was observed using any of these acids/bases. Interestingly, **2a** was converted into **3a** in very low yield when 1.2 equiv of *n-*Bu_4_N^+^F^−^ was used, despite *n-*Bu_4_N^+^F^−^ itself being less basic than the other bases (Scheme 2a) [42–43]. These results suggest that the properties of the counter cation may be important for the intramolecular cyclization of **2a**. Based on this observation, we next examined *n-*Bu_4_N^+^OH^−^, a more-basic R_4_N^+^X^−^ system, for the ring-closure of **2a**. Surprisingly, the reaction proceeded smoothly to give α-bromo-δ-valerolactone (**3a**) in 52% yield in 43 h (Scheme 2b). However, further extending the reaction time to 72 h resulted in a dramatically lower yield of **3a** (8%), most likely because **3a** is unstable to base at room temperature and may decompose or polymerize (see Supporting Information File 1).

To avoid decomposition or polymerization, **3a** produced in situ by the intramolecular cyclization of **2a** should be separated immediately from the reaction mixture containing *n-*Bu_4_N^+^OH^−^. Tetraalkylammonium salts are used as phase-transfer catalysts as they are soluble in both organic solvents and water. With these properties in mind, we next investigated the ring-closure of **2a** using a two-phase CHCl_3_/H_2_O system (Table 2). Intramolecular cyclization of the salt forms **3a**, which is extracted into the organic layer due to its low solubility in water. Because *n-*Bu_4_N^+^OH^−^ is less-soluble in organic solvents than water, **3a** is phase-separable from the base. To our delight, **2a** was smoothly converted into **3a** in 74% yield in this two-phase system, with the reaction time successfully reduced to 1 h (Table 2, entries 1–5). The use of a co-solvent to increase the solubility of **2a** was investigated in detail; DMSO was found to be the most effective solvent, with **3a** produced in 82% yield (Table 2, entries 5–12).

**Table 2:** Optimizing the intramolecular cyclization of **2a** in a two-phase system^a^.

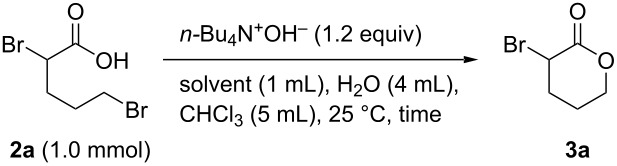			

Entry	Solvent	Time (h)	Yield **3a** (%)

1	CH_3_CN	24	29
2	CH_3_CN	18	38
3	CH_3_CN	9	58
4	CH_3_CN	3	65
5	CH_3_CN	1	74
6	MeOH	1	29
7	EtOH	1	51
8	iPrOH	1	68
9	THF	1	61
10	DMSO	1	82
11	DMF	1	64
12	none	1	65

^a^Yields were determined by ^1^H NMR spectroscopy using 1,3,5-trioxane as an internal standard.

We next optimized the base used to cyclize **2a** in the two-phase system under the optimized conditions (entry 10, Table 2), the results of which are summarized in Table 3. The use of tetraalkylammonium hydroxides with longer alkyl chains tended to increase the yield of **3a** (Table 3, entries 1–5), while the use of diisopropylethylamine, triethylamine, DBU, or Cs_2_CO_3_ was less effective (Table 3, entries 6–9); furthermore, the reaction did not proceed in the absence of a base (Table 3, entry 10). This investigation revealed that medium-chain tetraalkylammonium hydroxides, namely *n*-Bu_4_N^+^OH^−^ and *n*-Pr_4_N^+^OH^−^, effectively transform **2a** into **3a** through intramolecular cyclization.

**Table 3:** Optimizing the base for the intramolecular cyclization of **2a** to **3a**^a^.

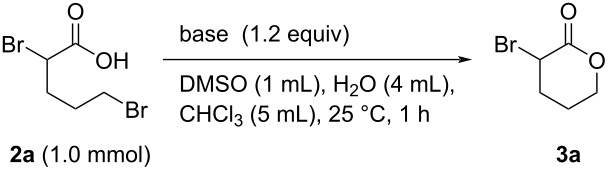		

Entry	Base	Yield **3a** (%)

1	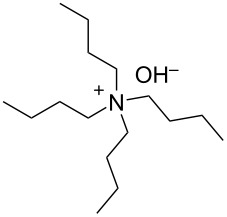 *n*-Bu_4_N^+^OH^–^ (40% in H_2_O)	82
2	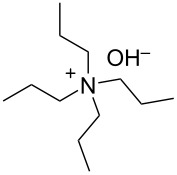 *n*-Pr_4_N^+^OH^–^ (40% in H_2_O)	67
3	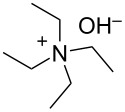 Et_4_N^+^OH^–^ (40% in H_2_O)	36
4	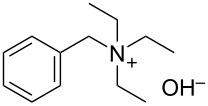 (10% in H_2_O)	37
5	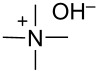 Me_4_N^+^OH^–^ (40% in H_2_O)	21
6	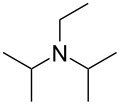	34
7	Et_3_N	34
8	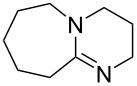	39
9	Cs_2_CO_3_	15
10	none	–

^a^Yields were determined by ^1^H NMR spectroscopy using 1,3,5-trioxane as an internal standard.

We next investigated the effect of the size of the lactone ring on the α-bromination reaction in this two-phase system. Table 4 shows that **2a** and **2b** were transformed to **3a** and **3b** in good yields, but **2c** did not react under these conditions due to the entropic cost associated with forming a seven-membered ring. Lactones **3a** and **3b** were obtained in good yields even when *n*-Pr_4_N^+^OH^−^ was used as the base [44].

**Table 4:** Reaction scope for the the intramolecular cyclization of **2** in a two-phase system^a^.

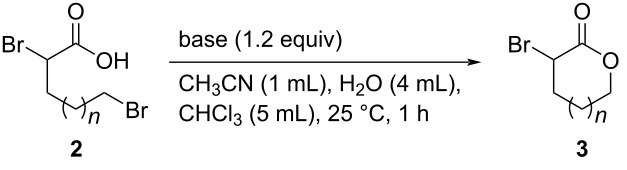			

Entry	*n*	Base	Yield **3** (%)

1	**2a**: *n* = 1	*n*-Bu_4_N^+^OH^−^	**3a**: 74
		*n*-Pr_4_N^+^OH^−^	**3a**: 75^b^
2	**2b**: *n* = 0	*n*-Bu_4_N^+^OH^−^	**3b**: 69
		*n*-Pr_4_N^+^OH^−^	**3b**: 73 (61)
3	**2c**: *n* = 2	*n*-Bu_4_N^+^OH^−^	**3c**: trace
		*n*-Pr_4_N^+^OH^−^	**3c**: trace

^a^Yields were determined by ^1^H NMR spectroscopy using 1,3,5-trioxane as an internal standard (isolated yield). ^b^See reference [44].

While the developed two-phase CHCl_3_/H_2_O system performed well for the syntheses of α-bromolactones, the use of CHCl_3_ as the reaction solvent should ideally be avoided because it is toxic and an environmental pollutant. Therefore, we further optimized the solvent combination to construct an eco-friendlier reaction system (Table 5). Various solvents were used as the organic layer instead of CHCl_3_, with **3a** produced in good yield using methyl ethyl ketone (MEK) as the solvent (Table 5, entries 1–5). Although the use of other ketones as solvents also afforded **3a** in moderate yields, MEK proved to be the most suitable replacement for CHCl_3_ (Table 5, entries 5–8).

**Table 5:** Optimizing the organic solvent in the two-phase system^a^_._

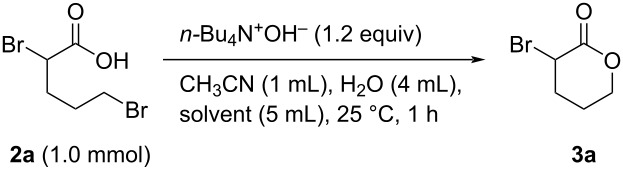		

Entry	Solvent	Yield **3a** (%)

1	CHCl_3_	74
2	FC-72	56
3	BTF	43
4	2-bromopropane	33
5	MEK	75
6	acetylacetone	64
7	3-methyl-2-butanone	55
8	pinacolone	49

^a^Yields were determined by ^1^H NMR spectroscopy using 1,3,5-trioxane as an internal standard.

**Scheme 3:**
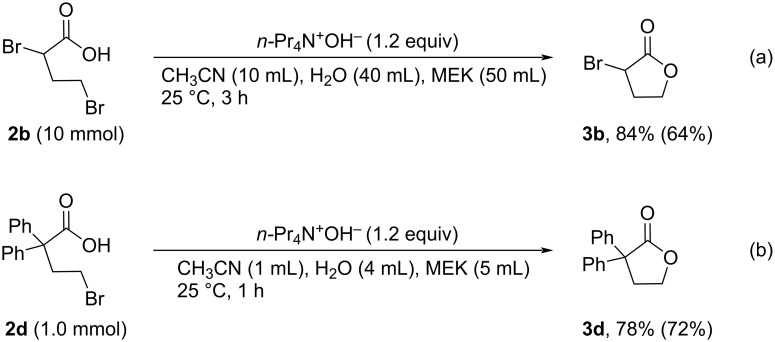
Synthesis of α*-*functionalized lactones using the two-phase system.

This environmentally friendly procedure was used to synthesize other lactones. For instance, this method was used to prepare **3b** from **2b** in 84% yield on a 10 mmol scale (Scheme 3a). Furthermore, this system also provided 2,2-diphenyl-γ-butyrolactone (**3d**), which bears two phenyl groups at the α-position, in 78% yield (Scheme 3b).

**Scheme 4:**
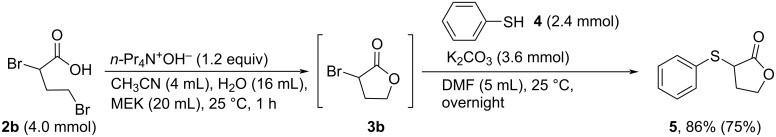
Synthesis of unsymmetrically functionalized sulfide **5** via the two-phase system-promoted intramolecular cyclization of **2b**.

α-Bromolactones were obtained without any handling difficulties using our developed system, with synthesis scale-up tolerated under mild conditions. To facilitate the construction of various functional scaffolds using this system, lactones were subsequently α-functionalized via the corresponding α-bromolactones using this two-phase system. Interestingly, **3b** synthesized using this method was smoothly substituted at the α-position with benzenethiol (**4**) in the presence of K_2_CO_3_ to afford the unsymmetrically functionalized sulfide **5** in 86% yield; **5** is a precursor to some pharmaceutical cores (Scheme 4) [45–48].

**Scheme 5:**
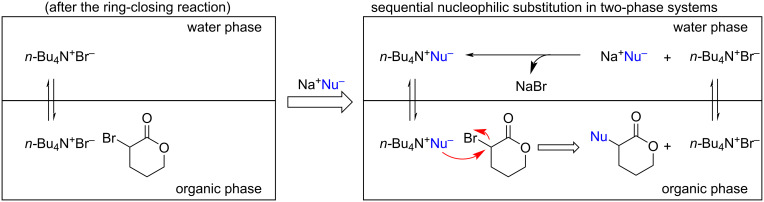
Sequential nucleophilic substitution in the two-phase system.

However, α-bromo-δ-valerolactone (**3a**) was extremely unstable under ambient conditions, and its purity quickly deteriorated even when stored in a freezer with shading (see Supporting Information File 1), resulting in trace amounts of α-functionalized lactones using the above-mentioned two-step method. Hence, we focused on sequential nucleophilic substitution in a two-phase system based on this bromination protocol. After ring-closing **2a**, the generated *α-*bromolactone **3a** was extracted into the organic layer, whereas the formed *n-*Bu_4_N^+^Br^−^ dissolved in both the aqueous and organic layers. *N*-Bu_4_N^+^Nu^−^ is formed when Na^+^Nu^−^ (Nu^−^: nucleophile) is added to the reaction mixture, which is then phase-transferred into the organic phase, with subsequent nucleophilic substitution at the α-position of **3a** proceeding directly to produce a variety of α-substituted lactones, along with the regeneration of *n*-Bu_4_N^+^Br^−^ (Scheme 5).

**Scheme 6:**
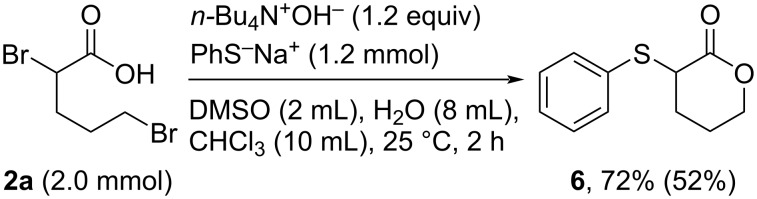
One-pot synthesis of 2-phenylthio-*α*-valerolactone **6**.

To demonstrate the applicability of this protocol, we investigated the synthesis of 2-phenylthio-α-valerolactone (**6**). Carboxylic acid **2a** (2.0 mmol) directly reacted with *n-*Bu_4_N^+^OH^−^ (1.2 equiv) and PhS^−^Na^+^ in the two-phase system under the optimized conditions for the synthesis of α-bromolactones, and **6** was successfully obtained in 72% yield (Scheme 6). These results demonstrate that this novel system facilitates the easy syntheses of functional molecules via α-bromolactones as key synthetic intermediates.

## Conclusion

In this study, we developed a facile and efficient method for α-brominating lactones using tetraalkylammonium hydroxide (R_4_N^+^OH^−^) as the base under mild conditions. Lactones were ring-opened with Br_2_ and a substoichiometric amount of PBr_3_, which led to the corresponding α-bromocarboxylic acids in good yields. These carboxylic acids subsequently underwent intramolecular cyclization in 1 h using a two-phase system (H_2_O/CHCl_3_) with R_4_N^+^OH^−^ to afford α-bromolactones in excellent yields. The use of methyl ethyl ketone (MEK) in the two-phase system led to an eco-friendly system amenable to large-scale synthesis. Furthermore, the α-bromolactones generated in situ by this method were transformed into functional molecules, such as α-thiolated lactones, in good yields without any handling difficulties. We expect that this new bromination system will lead to the use of various α-bromolactones as synthetic intermediates in organic chemistry.

## Experimental

**General comments.** Unless otherwise stated, all starting materials and catalysts were purchased from commercial sources and used without further purification. All solvents were used without distillation. ^1^H NMR spectra were recorded on a JEOL JNM-ECS400 (400 MHz) FT NMR system or a JEOL JNM-ECX400 (400 MHz) FT NMR system in CDCl_3_ with Me_4_Si as an internal standard. ^13^C{^1^H} NMR spectra were recorded on a JEOL JNM-ECX400 (100 MHz) FT NMR or JEOL JNM-ECS400 (100 MHz) FT NMR system in CDCl_3_.

**Ring-opening reaction of δ-valerolactone (1a) with Br_2_ in the presence of a catalytic amount of PBr_3_** (entry 1, Table 1). To a 50 mL three-neck flask were added δ-valerolactone (**1a**, 5 mmol) and PBr_3_ (5 mol %), then Br_2_ (1.0 equiv) was added dropwise for 2 h at 0 °C. After adding Br_2_, another amount of Br_2_ (1.0 equiv) was added to the reaction mixture for 30 min at 70 °C. The resulting solution was then stirred for 24 h at 80 °C. After the reaction was completed, the mixture was dissolved in CH_3_CN (30 mL) and bubbling N_2_ gas to remove excess amount of Br_2_ and the formed HBr (under open-air) then filtered. The filtrate was concentrated under reduced pressure to produce 2,5-dibromopentanoic acid **2a** in 90% yield with trace amount of **1a**. The purity of **2a** was determined by ^1^H and ^13^C NMR spectroscopy, and **2a** was used for the subsequent intramolecular cyclization without any further purification.

**General procedure for the synthesis of α-substituted lactones 3 via intramolecular cyclization of 2 with R_4_N^+^OH^−^ in two-phase system** (Table 4, Table 5 and Scheme 3b). To a 30 mL flask were added **2** (1.0 mmol, **2a**–**c**: synthesized and used without further purification; **2d**: purchased from commercial sources), CH_3_CN (1.0 mL), H_2_O (4 mL), CHCl_3_ (5.0 mL) or MEK (5.0 mL), and R_4_N^+^OH^−^ (1.2 equiv in aqueous solution). The mixture was stirred vigorously at 25 °C for 1 h. After the reaction was completed, the mixture was extracted with CHCl_3_ (15 mL × 3). The organic layer was washed with H_2_O (10 mL × 2), dried by anhydrous Na_2_SO_4_, then filtered. The filtrate was concentrated under reduced pressure. Finally, the residue was purified by gel permeation chromatography (eluent: CH_2_Cl_2_) or distillation to give pure product **3**.

**Gram-scale synthesis of α-bromolactone 3b in two-phase system with *n*-Pr_4_N^+^OH^−^** (Scheme 3a). To a 300 mL flask were added **2b** (10 mmol, synthesized by the procedure above mentioned and used without further purification), CH_3_CN (10 mL), H_2_O (40 mL), MEK (50 mL), and *n*-Pr_4_N^+^OH^−^ (1.2 equiv in aqueous solution). The mixture was stirred vigorously at 25 °C for 3 h. After the reaction was completed, the solvent was removed under reduced pressure. The residue was extracted with CHCl_3_ (20 mL × 3). The organic layer was washed with H_2_O (10 mL × 2), dried by anhydrous Na_2_SO_4_, then filtered. The filtrate was concentrated under reduced pressure. Finally, the residue was purified by distillation to give pure product **3b** in 64% yield (1.05 g).

**Cascade synthesis of 5 via two-phase ring closing of 3b and following substitution with benzenethiol 4 in the presence of K_2_CO_3_** (Scheme 4). To a 100 mL flask were added **2b** (4.0 mmol), CH_3_CN (4 mL), H_2_O (16 mL), MEK (20 mL), and *n*-Pr_4_N^+^OH^−^ (1.2 equiv in aqueous solution). The mixture was stirred vigorously at 25 °C for 1 h. After the reaction was completed, the mixture was extracted with CHCl_3_ (15 mL × 3). The organic layer was washed with H_2_O (10 mL × 2), dried by anhydrous Na_2_SO_4_, then filtered. The filtrate was concentrated under reduced pressure to give crude **3b**. To a 50 mL flask were added **3b** (used without isolation), benzenethiol **4** (2.4 mmol), DMF (5 mL), and K_2_CO_3_ (0.45 mmol), and the mixture was stirred at 25 °C overnight. The resulting mixture was extracted with CH_2_Cl_2_ (15 mL × 3). The organic layer was washed with H_2_O (10 mL × 2), dried by anhydrous Na_2_SO_4_, then filtered. The filtrate was concentrated under reduced pressure. Finally, the residue was purified by silica-gel column chromatography (AcOMe/isohexane) to give pure product **5**.

**One-pot synthesis of a functional lactone 6 using PhS^−^Na^+^ as the nucleophiles in the two-phase system** (Scheme 6). To a three-necked flask were added **2a** (2.0 mmol), DMSO (2 mL), H_2_O (4 mL), CHCl_3_ (10 mL), and *n*-Bu_4_N^+^OH^−^ (1.2 equiv in aqueous solution), and stirred vigorously for 10 min at 25 °C. Then, a solution of PhS^−^Na^+^ (1.2 mmol) in H_2_O (4 mL) was slowly added over 30 min. After the addition, the solution was further stirred for 80 min. The resulting mixture was extracted with Et_2_O (15 mL × 3), and the organic layer was washed with 1% HCl aq (10 mL), H_2_O (10 mL × 2), and dried by anhydrous MgSO_4_. The filtration was carried out, and the filtrate was concentrated under reduced pressure. Finally, the residue was purified by silica-gel column chromatography (Et_2_O/isohexane) to give pure product **6**.

## Supporting Information

File 1 Evaluation of the stability of *α*-bromo-*δ*-valerolactone (**3a**, Table S1), characterization data of compounds (**3a**, **3b**, **3d**, **5**, and **6**), and copies of ^1^H NMR and ^13^C{^1^H} NMR spectra.
