# Supplementary material for: A two-phase bromination process using tetraalkylammonium hydroxide for the practical synthesis of α-bromolactones from lactones
Source: Beilstein J Org Chem. 2021 Dec 9;17:2906–14. doi: 10.3762/bjoc.17.198 (PMC8685563; doi:10.3762/bjoc.17.198)

BEILSTEIN JOURNAL OF ORGANIC CHEMISTRY

Supporting Information

for

A two-phase bromination process using tetraalkylammonium hydroxide for the practical synthesis of α-bromolactones from lactones

Yuki Yamamoto, Akihiro Tabuchi, Kazumi Hosono, Takanori Ochi, Kento Yamazaki, Shintaro Kodama, Akihiro Nomoto and Akiya Ogawa

Beilstein J. Org. Chem. **2021,** *17,* 2906–2914. doi:10.3762/bjoc.17.198

Evaluation of the stability of *α*-bromo-*δ*-valerolactone (3a, Table S1), characterization data of compounds (3a, 3b, 3d, 5, and 6), and copies of ^{1}H NMR and ^{13}C{^{1}H} NMR spectra

License and Terms: This is a supporting information file under the terms of the Creative Commons Attribution License (https://creativecommons.org/licenses/by/4.0). Please note that the reuse, redistribution and reproduction in particular requires that the author(s) and source are credited and that individual graphics may be subject to special legal provisions.
The license is subject to the *Beilstein Journal of Organic Chemistry* terms and conditions: (https://www.beilstein-journals.org/bjoc/terms)

CONTENTS

page

Table S1. Evaluation of the stability of *α*-bromo-*δ*-valerolactone **3a** S2

Characterization data of the compounds **3a**, **3b**, **3d**, **5**, and **6** S2–S3

Copies of ^{1}H NMR and ^{13}C NMR spectra of compounds **3a**, **3b**, **3d**, **5**, and **6** S4–S8

Table S1. Evaluation of the stability of *α*-bromo-*δ*-valerolactone **3a**

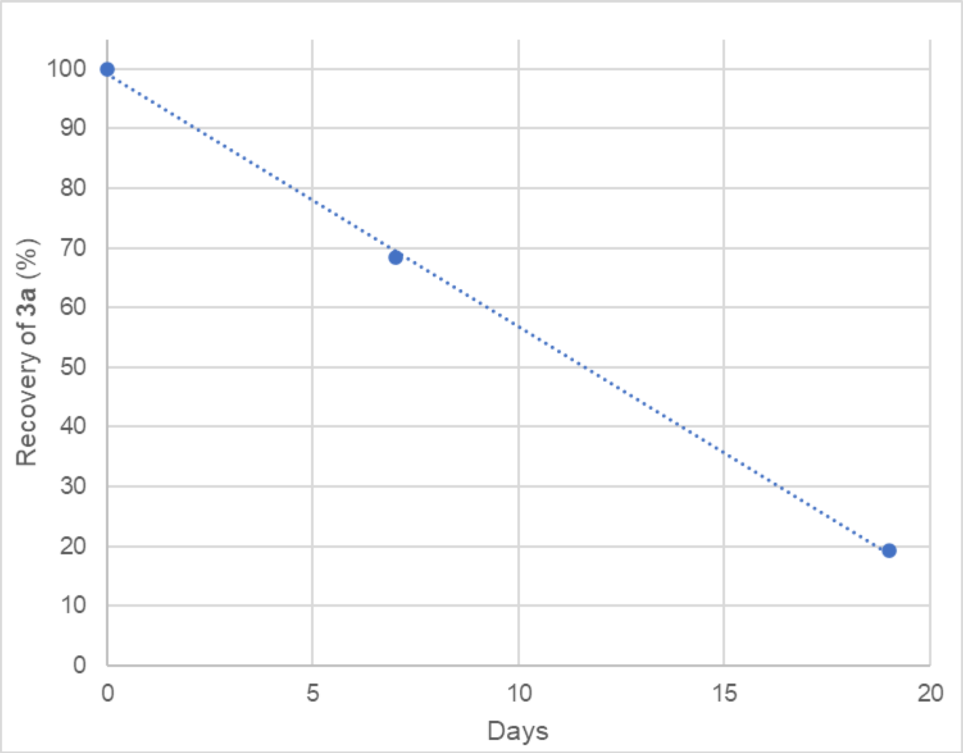

[Method] Initially, **3a** was synthesized following the general procedure (eq 1).

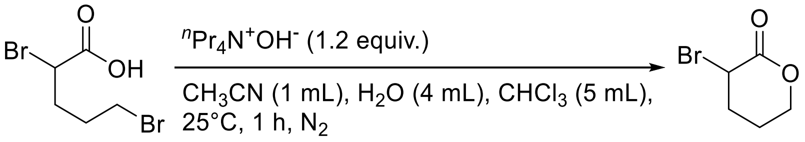

(1)

2a (1.0 mmol) **3a** (73%)

After extracting with $CHCl_3$, washing with H_2O, and removing the solvent under reduced pressure, the crude mixture of **3a** was stored at -30 °C (in a freezer) with shading. The purity of **3a** (i.e. the relative recovery of **3a** based on the initial production) was confirmed by ^1H NMR spectroscopy using 1,3,5-trioxane as the internal standard (solvent: $CDCl_3$).

・**Characterization data**

3-Bromotetrahydro-2*H*-pyran-2-one (3a, entry 1 in Table 4). [CAS no. 55974-69-1].[1] Purified by gel permeation chromatography (see the reference [44]), colorless oil, 35.2

mg, 20%; ^{1}H NMR (400MHz, $CDCl_3$): δ 4.62-4.56 (m, 2H), 4.44-4.38 (m, 1H), 2.52-2.43 (m, 1H), 2.39-2.20 (m, 2H), 1.95-1.86 (m, 1H); ^{13}C{^{1}H} NMR (100 MHz, $CDCl_3$): δ 166.9, 70.0, 40.9, 30.3, 20.0.

3-Bromodihydrofuran-2(3*H*)-one (3b, entry 2 in Table 4). [CAS no. 5061-21-2].[2] Light yellow oil, 101.5 mg, 61%; ^{1}H NMR (400MHz, $CDCl_3$): δ 4.55-4.49 (m, 1H), 4.48-4.42 (m, 2H), 2.89-2.79 (m, 1H), 2.55-2.48 (m, 1H); ^{13}C{^{1}H} NMR (100 MHz, $CDCl_3$): δ 173.3, 67.1, 37.7, 33.7.

3,3-Diphenyldihydrofuran-2(3*H*)-one (3d, Scheme 3b). [CAS no. 956-89-8].[3] White solid, 170.9 mg, 72%; ^{1}H NMR (400 MHz, $CDCl_3$): δ 7.34-7.21 (m, 10H), 4.19 (t, J = 6.3 Hz, 2H), 2.91 (t, J = 6.6 Hz, 2H); ^{13}C{^{1}H} NMR (100 MHz, $CDCl_3$): δ 177.9, 140.8, 128.8, 127.7, 127.6, 65.4, 56.5, 37.6.

3-(Phenylthio)dihydrofuran-2(3*H*)-one (5, Scheme 4). [CAS no. 35998-30-2].[4] Light yellow oil, 349.1 mg, 75%; ^{1}H NMR (400 MHz, $CDCl_3$): δ 7.53-7.51 (m, 2H), 7.33-7.31 (m, 3H), 4.24-4.14 (m, 2H), 3.89-3.85 (m, 1H), 2.68-2.59 (m, 1H), 2.26-2.17 (m, 1H); ^{13}C{^{1}H} NMR (100 MHz, $CDCl_3$): δ 175.3, 133.4, 132.0, 129.4, 128.8, 128.7, 128.5, 66.7, 44.4, 30.0.

3-(Phenylthio)tetrahydro-2*H*-pyran-2-one (6, Scheme 5). [CAS no. 89036-08-8].[5] Light yellow oil, 129.9 mg, 52%; ^{1}H NMR (400 MHz, $CDCl_3$): δ 7.55-7.49 (m, 2H), 7.37-7.27 (m, 3H), 4.45-4.29 (m, 2H), 3.92 (t, J = 7.0 Hz, 1H), 2.35-2.26 (m, 1H), 2.06-1.97 (m, 2H), 1.91-1.81 (m, 1H); ^{13}C{^{1}H} NMR (100 MHz, $CDCl_3$): δ 169.9, 133.4, 132.9, 129.3, 128.5, 69.2, 46.7, 26.7, 21.3.

・References

1. Chavda, J.K.; Procopiou, P. A.; Horton, P. N.; Coles, S. J.; Porter, M. J. *Eur. J. Org. Chem.* **2014**, 129–139.
2. Yang, H.; Gao, Y.; Qiao, X.; Xie, L.; Xu, X. *Org. Lett.* **2011**, *13*, 3670–3673.
3. Bartalucci, N.; Marchetti, F.; Zacchini, S.; Pampaloni, G. *Dalton Trans.* **2019**, *48*, 5725–5734.
4. Chen, K.; Zhang, S-Q.; Brandenberg, O. F.; Hong, X.; Arnold, F. H. *J. Am. Chem. Soc.* **2018**, *140*, 16402–16407.
5. Kato, M.; Ouchi, A.; Yoshikoshi, A. *Bull. Chem. Soc. Jpn.* **1991**, *64*, 1479–1486.

Figure S1. ^{1}H and $^{13}C\{^{1}H\}$ NMR spectra of compound **3a**

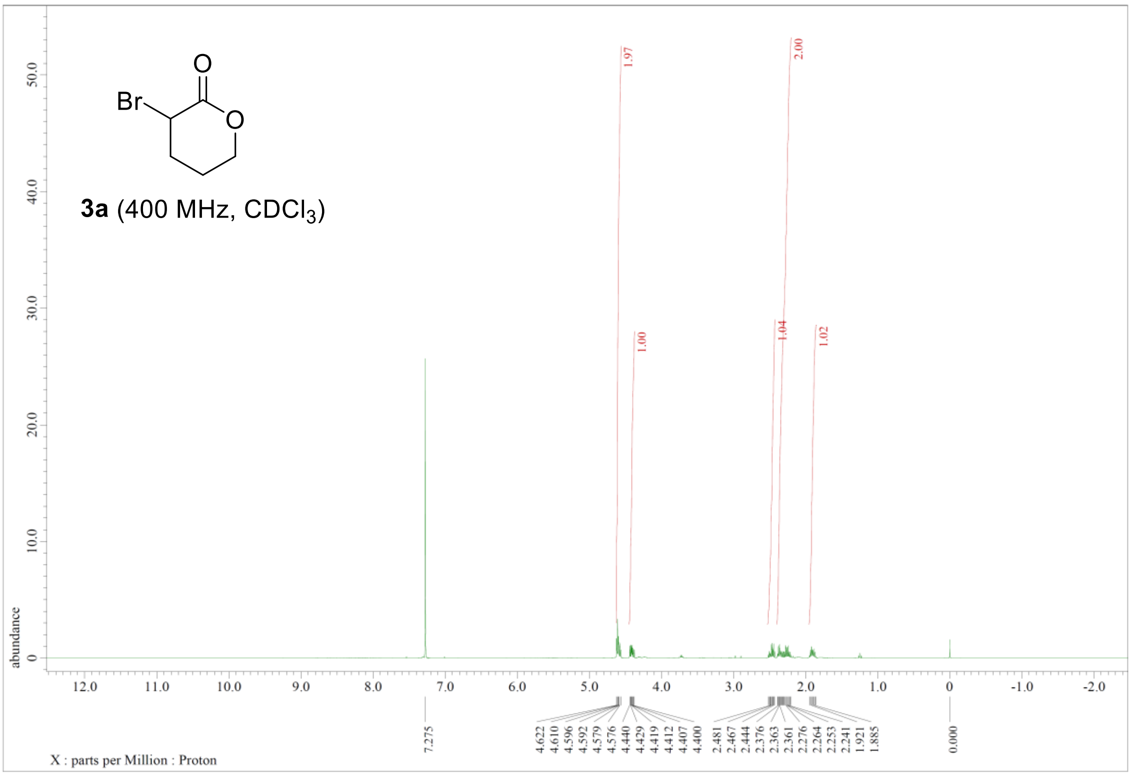

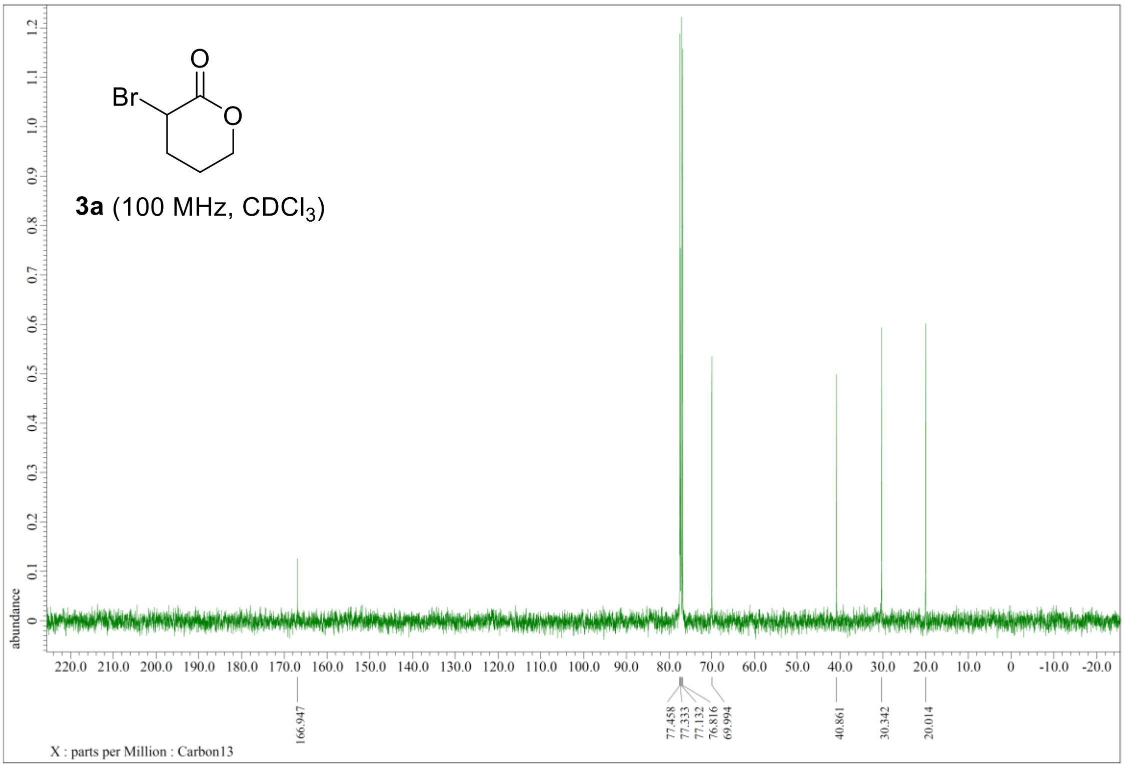

Figure S2. ^{1}H and $^{13}C\{^{1}H\}$ NMR spectra of compound **3b**

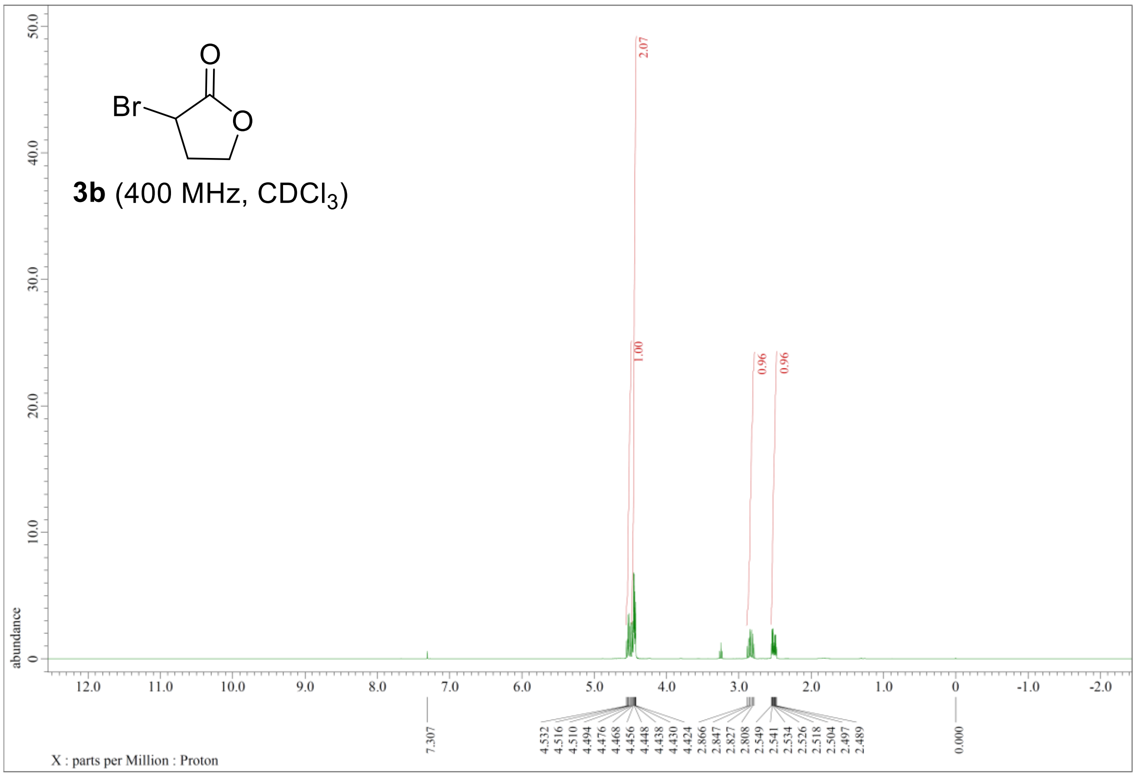

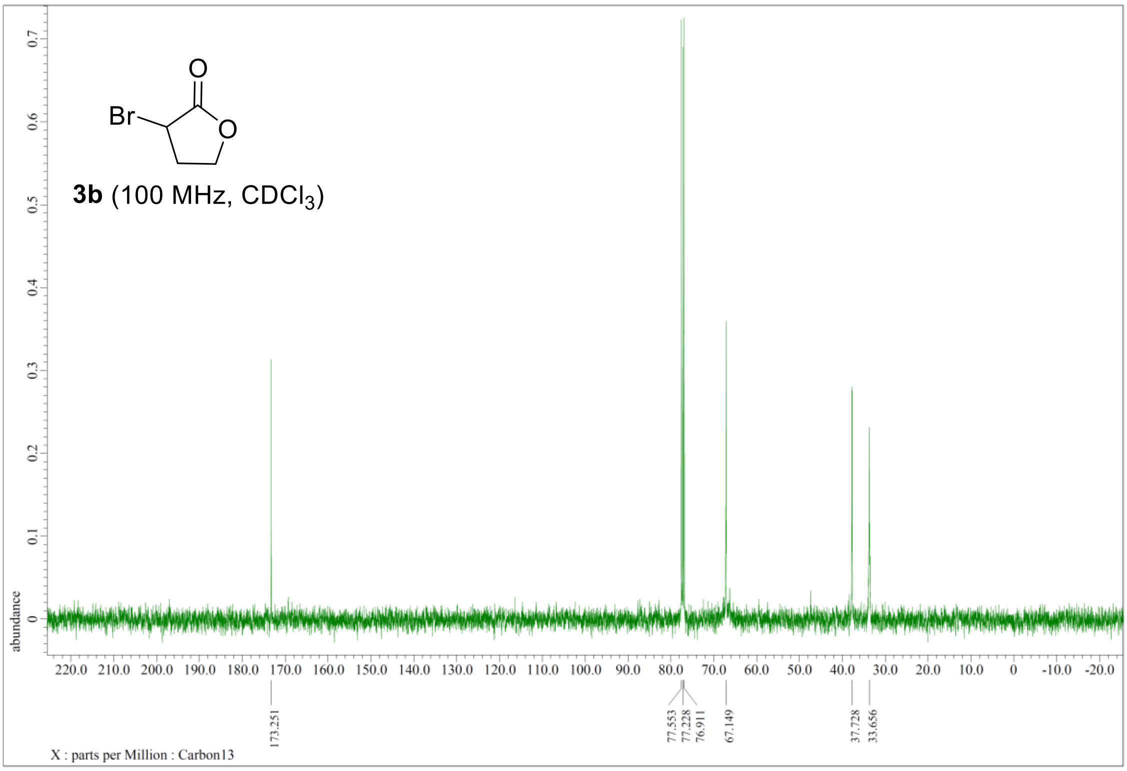

Figure S3. ^{1}H and $^{13}C\{^{1}H\}$ NMR spectra of compound **3d**

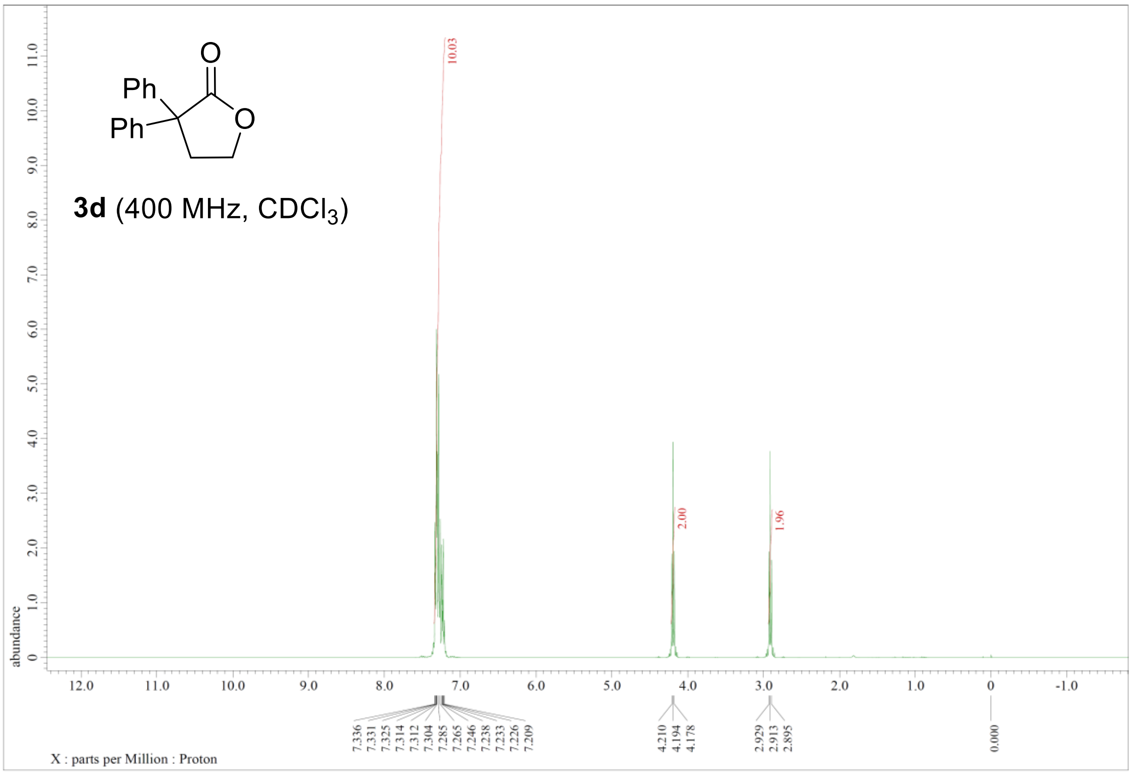

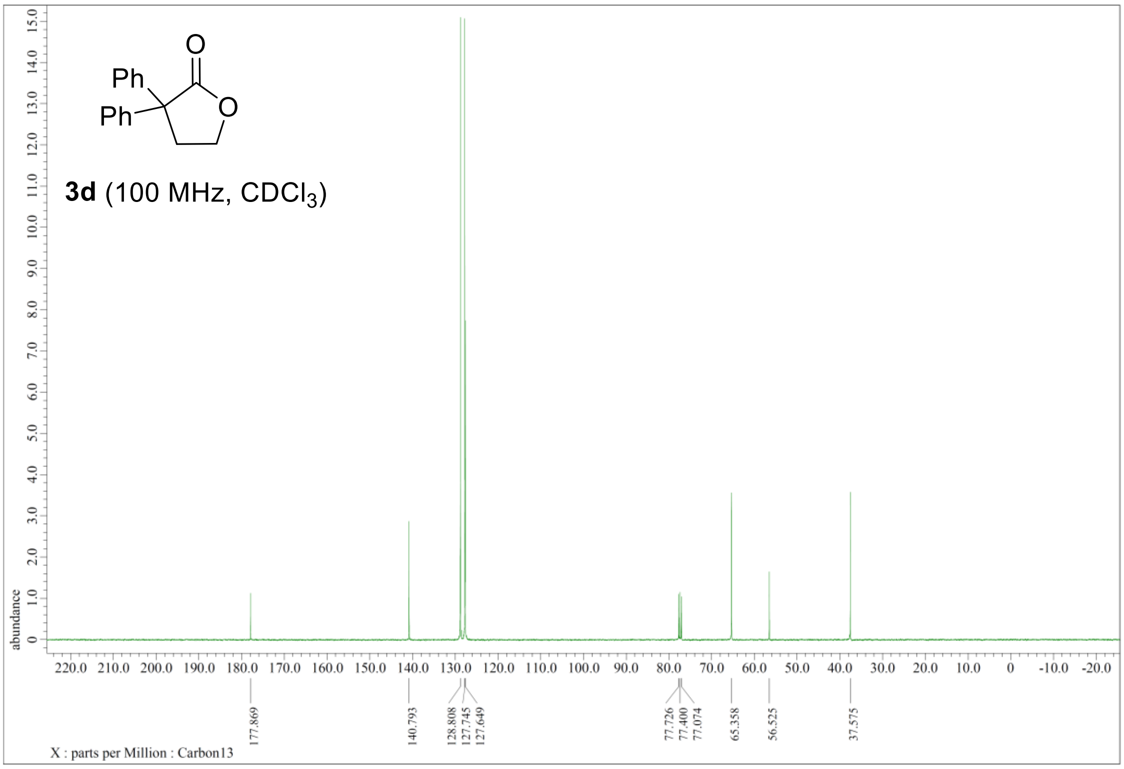

Figure S4. ^{1}H and $^{13}C\{^{1}H\}$ NMR spectra of compound **5**

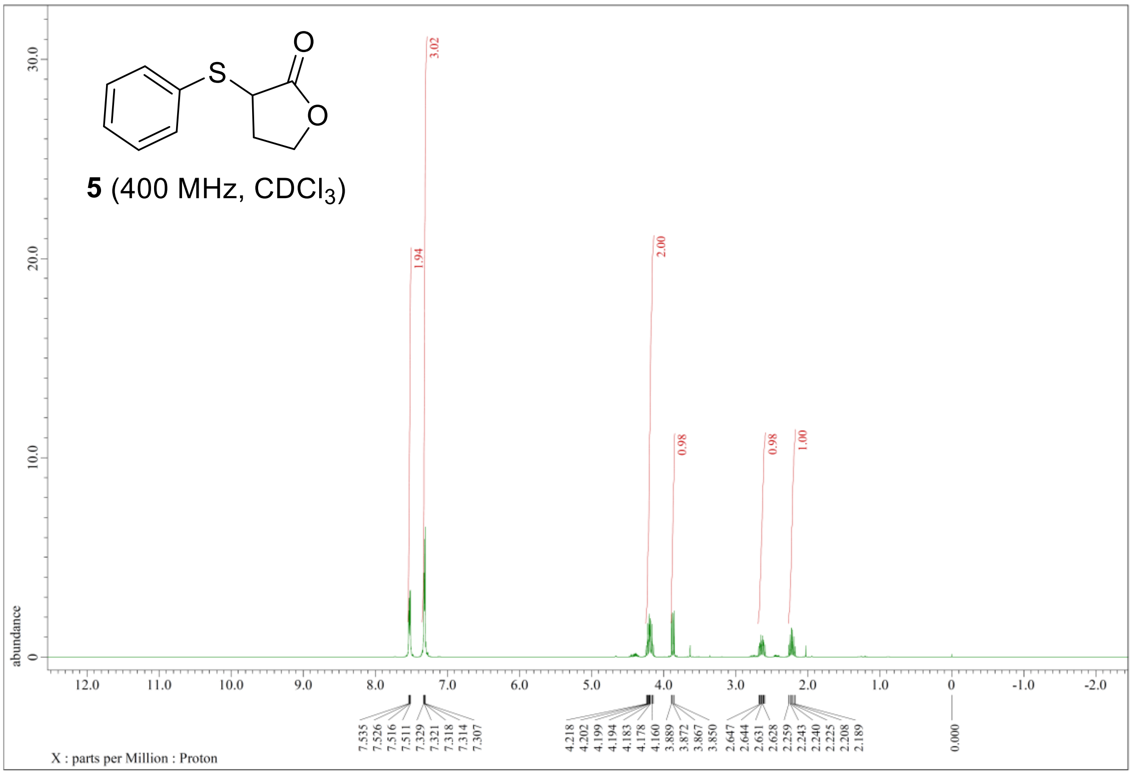

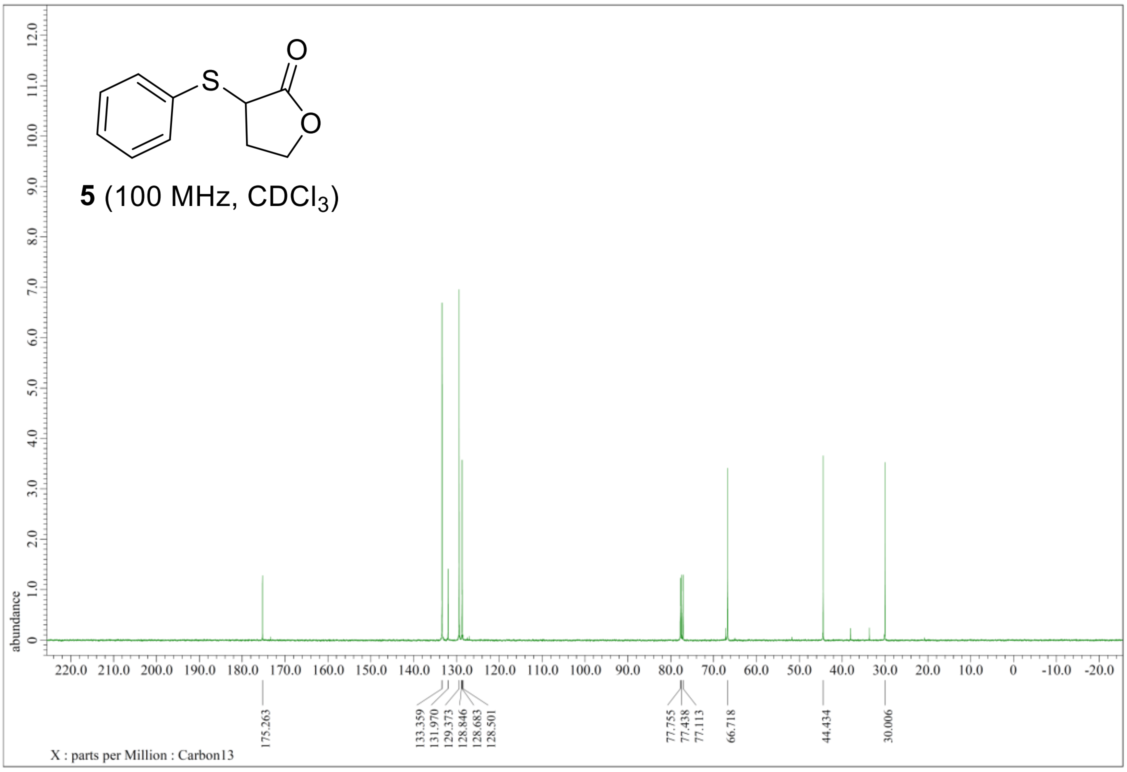

Figure S5. ^1H and $^{13}C\{^1H\}$ NMR spectra of compound **6**

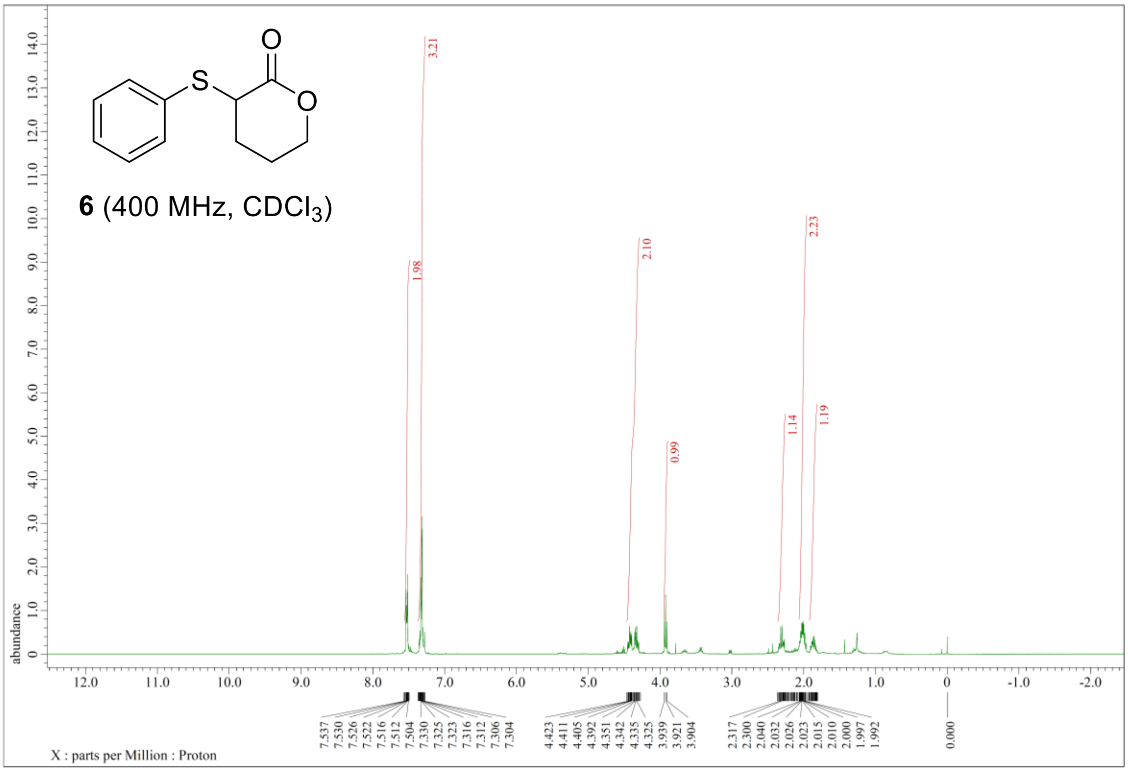

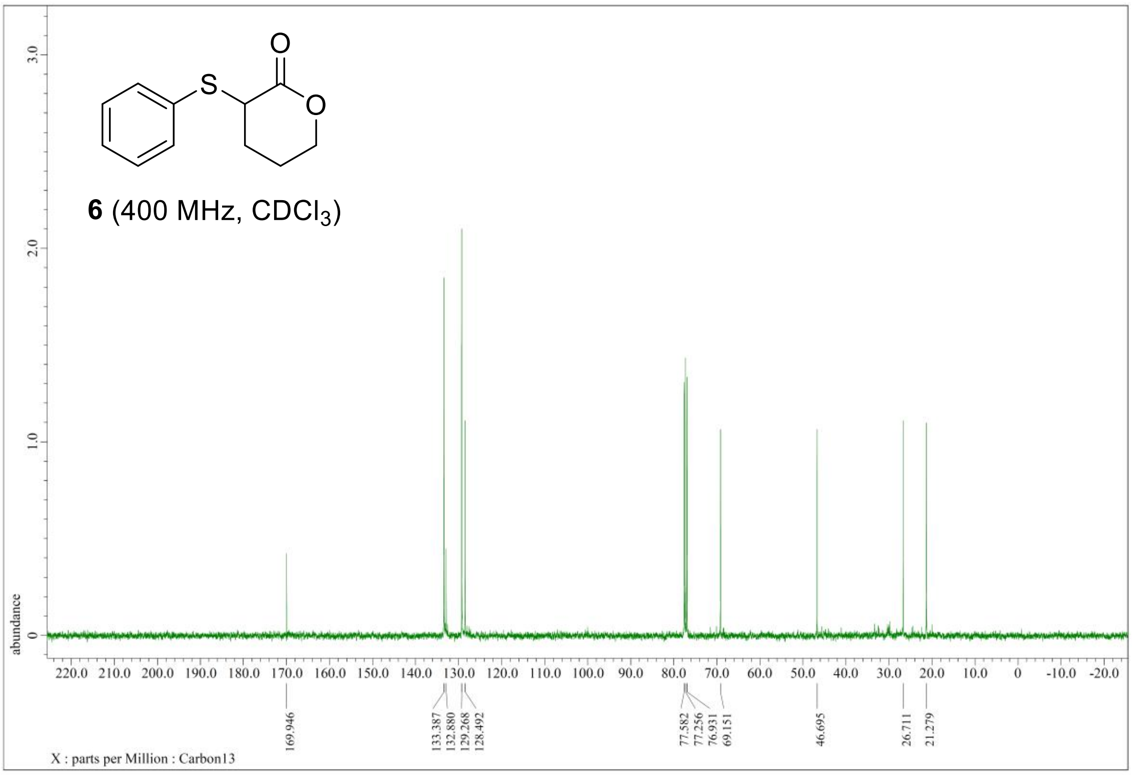

Supplement: File 1 — Evaluation of the stability of α-bromo-δ-valerolactone (3a, Table S1), characterization data of compounds (3a, 3b, 3d, 5, and 6), and copies of 1H NMR and 13C{1H} NMR spectra. [file Beilstein_J_Org_Chem-17-2906-s001.pdf]
